# Distinct subtypes of the negative hemodynamic response function across cortical distance and depth in human visual cortex

**DOI:** 10.21203/rs.3.rs-11088189/v1

**Published:** 2026-10-08

**Authors:** Artemy Vinogradov, Nooshin J. Fesharaki, Junseok Park, Eunyoung Lee, Huimahn A. Choi, Jung Hwan Kim

**Affiliations:** The University of Texas Health Science Center at Houston; The University of Texas Health Science Center at Houston; Texas Children’s Hospital; The University of Texas Health Science Center at Houston; The University of Texas Health Science Center at Houston; The University of Texas Health Science Center at Houston

## Abstract

The negative blood-oxygenation-level-dependent (BOLD) response (NBR) and its origins have been investigated for over a decade. However, the temporal characteristics and spatial organization of the NBR are not fully understood. Previous studies by our group and others have shown that the negative HRF (nHRF)—the NBR evoked by a brief stimulus—exhibits substantial spatial variability. To investigate this spatial variability in greater detail, we used a unilateral parafoveal visual stimulus and examine nHRF temporal dynamics as a function of cortical distance from the stimulus representation (SR) and throughout cortical depth in both the contralateral and ipsilateral visual cortex. We identified two temporally distinct patterns of nHRF dynamics and defined the corresponding subtypes: 1) a transient nHRF (T-nHRF), characterized by a weak but significant initial negative response (INR) followed by a prominent positive peak, and 2) a fully developed nHRF (FD-nHRF), characterized by a delayed and strong INR followed by a weak or absent positive peak. We observed that the two subtypes coexisted throughout visual cortex in regionally varying proportions. T-nHRFs predominated near the SR while FD-nHRFs predominated in more peripheral contralateral regions and homologous ipsilateral regions. Response magnitudes increased toward the superficial gray matter, with pronounced depth dependence in the positive peak for T-nHRFs and in the INR for FD-nHRFs. For each subtype, depth dependence was most consistent in regions where that subtype predominated. The regional distributions and depth-dependent response profiles of two nHRF subtypes showed systematic nHRF organization with respect to cortical distance from the SR and cortical depth. This organization suggests that distinct NBR mechanisms may contribute to the two subtypes, with their relative contributions varying cortical locations.

## 1. INTRODUCTION

Blood-oxygenation-level-dependent (BOLD) functional magnetic resonance imaging (fMRI) infers local neural activity through its associated hemodynamic changes, which provides a non-invasive means of mapping functional brain activity. Although it is an indirect measure that captures hemodynamic changes following changes in neural activity, it has had a major impact on human cognitive neuroscience because it non-invasively detects sensitive signals with high sensitivity in response to a stimulus or during spontaneous neuronal fluctuations^1,2^.

The negative BOLD response (NBR), the decrease in BOLD signal in regions adjacent to the positive BOLD response (PBR) and/or ipsilateral to a stimulus, has been frequently observed in BOLD fMRI experiments with different tasks (visual^3,4^, motor^5,6^, auditory^7,8^ and memory^8,9^) and stimulations (electrical nerve^10,11^ and tactile^12^). The NBR has been reported across diverse brain regions, with its spatial extent and localization. Previous studies have shown NBRs both locally, in regions adjacent to areas exhibiting PBRs^7^, and remotely, in the functionally homologous cortical regions of the hemisphere ipsilateral to a stimulus^13^. In addition, NBRs have been associated with broader network-level suppression, particularly within task-negative networks such as the default mode network (DMN)^8^. Accordingly, several mechanisms have been proposed to explain the NBR, including vascular steal effects^4,14^, neural suppression^11,15^, and non-neural vascular mechanisms^16,17^.

The PBR evoked by a brief (< 4 s) stimulus, commonly referred to as the positive hemodynamic response function (pHRF), exhibits stereotypical temporal dynamics: a rise to a peak followed by a post-stimulus undershoot before returning to baseline. The linearity between the pHRF and the underlying neural activity has been commonly assumed when interpreting task- or stimulus-based BOLD data^18^. The associated NBR, observed in regions adjacent to the pHRF and/or in hemisphere ipsilateral to the stimulus, has been termed the negative HRF (nHRF). In earlier studies, the nHRF has been reported to have similar temporal dynamics to an upside down version of the pHRF (the inverted pHRF)^4,7^. In our prior work, we precisely delineated the stimulus representation (SR) using retinotopic mapping to examine contralateral and ipsilateral nHRFs in early visual cortex^19^. Outside the SR in contralateral side, the nHRF exhibited a progressive temporal evolution with respect to distance from the SR; a weak initial negative response (INR) followed by a prominent positive peak near the SR to a strong INR followed by a weak positive peak in more distant regions. The nHRF, similar to the inverted pHRF, was observed in regions distant from the SR, which consistent with previous studies. In addition, we observed nHRFs with pronounced INRs accompanied by weak peaks in ipsilateral side.

Depth-dependent PBRs have been investigated across multiple cortical regions in both animals^3,20^ and humans, including the visual^21,22^, motor^23–25^, somatosensory^26^, and memory-related cortical regions^27^. In previous depth-dependent pHRF studies, larger and delayed peak responses were consistently observed in superficial gray matter compared to deep gray matter in various brain regions^28,29^. In contrast, only a few studies have examined depth-dependent NBRs, reporting stronger NBRs in superficial gray matter relative to deep gray matter^3,13^. However, a detailed characterization of depth-dependent temporal dynamics of the nHRF has not been fully explored.

In this study, we used a unilateral visual stimulus near the foveal representation to produce nHRFs and examined variations of temporal dynamics of the nHRF as a function of cortical depth on both contralateral and ipsilateral regions in visual cortex. To characterize details of nHRF dynamics, we introduced two different types of the nHRF—transient nHRF (T-nHRF) and Full-developed nHRF (FD nHRF). We, then examined each nHRF subtype in three different regions: 1) near the SR, 2) peripheral contralateral regions, and 3) homologous ipsilateral regions in the visual cortex to provide a detailed characterization of the nHRF.

## 2. METHODS

### 2.1 Participants

Ten healthy subjects (sex-balanced, aged 23–39 years, with a mean age of 29.3 years) with normal or corrected-to-normal visual acuity were recruited. A written, informed consent form was obtained for each subject at the time of first enrollment. All experimental procedures were conducted according to a protocol reviewed and approved by the Institutional Review Board of the University of Texas Health Science Center at Houston and based on the principles of the Belmont Report^30^. All participants were also screened for MRI safety and trained on task prior to the experiment.

### 2.2 MRI Protocol and preprocessing data

Imaging data was collected using a 3T MAGNETOM Prisma MRI scanner (Siemens Healthcare, Erlangen, Germany) equipped with a 64-channel RF head coil. Visual stimuli were created using MATLAB (The MathWorks, Inc.) with PsychToolbox-3^31^ and presented on a 32-inch BOLD screen (Cambridge Research Systems, Kent, United Kingdom) at the back of the scanner, which participants viewed through a mirror.

T2*-weighted functional data was obtained using an accelerated echo-planar imaging (EPI) sequence with 3x blipped-CAIPI simultaneous multi-slice (SMS) acceleration (GRAPPA = 2, TR = 1250ms, TE = 29ms, field of view (FOV) = 180mm, Ernst flip angle = 67°, 1.5-mm isotropic voxels, and 36 quasi-coronal slices perpendicular to the calcarine sulcus)^32,33^. Fast TopUp scans (~ 30 s) with reversed phase encoding were collected after each run, which were used to correct for susceptibility-induced magnetic field distortions^34^. Additionally, high-order shimming was applied every other functional scan to reduce off-resonance distortion effects. Following the functional scans, a low-resolution T1-weighted structural volume, referred to as the “Inplane” anatomy was also acquired with the same slice prescription as the functional data using a 3D FLASH sequence (minimum TR/TE, 1.1-mm slice thickness, FOV = 180, 1.5-mm inplane pixel size, flip angle = 10°, and 40 slices). The Inplane anatomy served as an intermediate reference for registering the functional data to a high-resolution structural volume. High-resolution T1-weighted structural images were acquired using an MP-RAGE sequence (TR = 2400ms, T1 = 1160ms, flip angle = 8°, and 0.7-mm isotropic voxels).

For the functional data, slice–timing correction was applied using the center slice as the temporal reference after removing the initial baseline period (the first 10 s of each scan). Motion correction was then performed to account for both within- and between-scan head motion using a robust expectation-maximization algorithm^35^. To reduce coil-related intensity inhomogeneities, the functional data were spatially normalized by dividing each volume by a temporally averaged, spatially smoothed intensity image with an additive correction for robustness^21^. Susceptibility-induced magnetic field distortion correction was subsequently applied to remediate low-frequency intensity nonuniformities arising from B0 and B1 field inhomogeneities^21^. Linear trends were also removed through temporal detrending. Finally, the corrected functional datasets were spatially aligned to the high-resolution structural volume using the Inplane anatomy collected in each session.

### 2.3 Stimulus

During each functional scan, subjects maintained their eyes on a central dot (0.24° diameter) while performing a slow event-related visual task consisting of repeated epochs of a 2-s visual stimulus followed by a 28-s a non-demanding, color-detection fixation task (Fig. 1A). Each scan began with a 10-s baseline period before the first trial to minimize transition effects. Trials were initiated by a 0.5-s black-on-white cue presented at fixation, followed by a 2-s visual stimulus designed to evoke a hemodynamic response within the SR of the visual cortex. The stimulus consisted of a partial annulus (eccentricity: 1–3°, polar angle: 50–130°) comprising ten high-contrast (85%) radial grating wedge-shaped sectors (1 cycle/degree) arranged in two concentric rings and counterphase flickering at 4 Hz. On 50% of trials, a randomly selected sector contained a contrast decrement (~ 21%), with the exact decrement adjusted for each subject to maintain task difficulty. Subjects indicated the presence or absence of the contrast decrement by pressing one of two buttons while maintaining central fixation. Following stimulus presentation, subjects performed a 28-s color-detection fixation task designed to maintain attention while minimizing task-related cognitive demands. During this period, the central dot changed color every 0.5 s among a set of 20 colors, and subjects pressed a button whenever a light-green dot appeared. Each functional scan contained 12 trials, and six scans were acquired per subject, yielding a total of 72 HRFs.

### 2.4 Retinotopy and visual regions-of-interest (ROIs)

Eccentricity and polar angle maps were generated using back-projection tomography^36^ in a separate session to delineate the SR corresponding to the visual stimulus for each subject. During retinotopy mapping, subjects maintained their eyes on a color-changing central dot and performed a non-demanding color-detection task while flickering checkerboard bars swept sequentially across the visual field in 12 directions separated by 15° increments. In a separate scan, the bars swept in the opposite direction, such that the forward and reverse scans together sampled the full 360° range of sweep directions. Two forward-reverse scan pairs were acquired.

For retinotopy reconstruction, the initial HRF was modeled using a canonical difference-of-gamma function^37^. The HRF was estimated separately from the averaged forward and reverse sweep data and then iteratively adjusted to minimize differences between the deconvolved responses from the two sweep directions. The optimized HRF was subsequently used to reconstruct retinotopic maps from all 24 sweep directions.

Subject-specific eccentricity and polar angle maps were generated from the reconstructed retinotopic data and projected onto a flattened representation of the visual cortex^38,39^. These maps were used to delineate V1 boundaries in both hemispheres based on its characteristic eccentricity and polar-angle organization. Using the V1 boundaries as a reference, dorsal and ventral V2/V3, as well as V3AB, were subsequently delineated according to their retinotopic organization relative to V1. The hV4 region was excluded because its substantial foveal representations overlapped extensively with the SR, making it unsuitable for nHRF analysis using our foveal stimulus. For each visual area, the contralateral SR ROI was defined as the cortical region corresponding to 1–3° eccentricity and 50–130° polar angle. Finally, all visual ROIs were projected from the flattened cortical representation back onto the native cortical surface for subsequent analyses.

### 2.5 Dilation ROIs

To investigate spatial variation in temporal dynamics of the nHRF along the cortical surface, we generated a series of band-shaped dilation ROIs extending outward from the SR using a flood-fill method^40^. Cortical surface distances from the SR toward the peripheral visual field representations were calculated within each visual area. The first dilation ROI was defined as a 1.5-mm-wide band immediately adjacent to the SR using vector analysis and extending outward toward the periphery. Then, we continued to generate 1.5-mm-wide dilation ROIs iteratively, producing a series of contiguous bands that spanned the cortical surface from the SR to the peripheral representations. Foveal regions were excluded from the analysis to focus on changes in nHRF dynamics with increasing distance from the SR.

### 2.6 Depth coordinate system

High-resolution anatomical volumes were segmented using FreeSurfer^41^. The gray–white matter interface (S_gw_) was generated using iso-surface contour extraction followed by mesh refinement using a volume-preserving deformable-mesh algorithm. The pial surface (S_p_) mesh was obtained directly from the FreeSurfer segmentation. Signed-distance functions (SDFs) were calculated from these surfaces. A constrained dilation algorithm was first applied to the high-resolution anatomical volume to obtain connected representations of gray matter nodes. For each node, Euclidean distances to both cortical surfaces were calculated, yielding SDF_gw_ (positive within the gray matter relative to S_gw_) and SDF_p_ (negative within gray matter relative to S_p_). Cortical depth was defined using a normalized depth coordinate, *w* = (SDF_gw_ / SDF_gw_ – SDF_p_), where *w* = 0 at the gray–white matter interface and *w* = 1 at the pial surface. The normalized depth was computed analytically in most cases; however, a root-solving algorithm was used when the denominator was close to zero^42^.

Laminar streamlines were generated to associate gray matter nodes across cortical depths by calculating trajectories through intermediate layers between S_gw_ and S_p_ using a five-stencil gradient method. This approach provides a self-reciprocal depth coordinate system that is independent of local cortical thickness and preserves one-to-one correspondence between vertices on S_gw_ and S_p_^43–45^.

### 2.7 HRF analysis

For HRF analysis, the mean HRF and its standard error of the mean (SEM) from 72 events and subsequently upsampled to a temporal resolution of 0.1 s using piecewise cubic Hermite interpolation. Baseline correction was performed by subtracting the average of the first and last time points from each HRF. Four parameters were then extracted from the upsampled nHRFs (Fig. 1B). Peak amplitude was defined as the maximum response occurring more than 2 s after stimulus onset, and the time-to-peak (TTP) was defined as the corresponding latency. The INR was defined as the largest negative deflection preceding the peak (if present), with the time-to-INR (TTI) defined as its corresponding latency.

To assess the reliability of nHRFs, we calculated the contrast-to-noise ratio (CNR) for each voxel as the ratio of the absolute INR amplitude to its SEM. Voxels with CNR > 1 were considered to exhibit significant nHRFs. An analogous procedure was used to identify significant pHRFs within the SR, based on the peak amplitude and its SEM.

To further quantify nHRF temporal characteristics, we performed coherence analysis. For each voxel, a sinusoid at the stimulus frequency (1/30 Hz) was fit to the mean time series, yielding a coherence value that quantified the strength of stimulus-locked oscillatory responses. Voxels with coherence values > 0.10 were retained for subsequent analyses to ensure reliable estimation of temporal dynamics.

### 2.8 Cortical distance and depth analyses

Functional data were averaged across cortical depth corresponding to normalized depth values (*w*) between 0.2 and 0.8 along the laminar streamlines to minimize partial–volume effects. The resulting depth-averaged time series were then projected onto the corresponding gray–white matter interface for surface-based analyses.

To estimate variability in the nHRF, bootstrapping was performed by resampling with replacement across 72 events and repeating the procedure 2,000 times. The mean bootstrapped nHRFs and their 68% confidence interval (CI), which corresponds approximately to ± 1 SEM under the assumption of normality, were then computed^46,47^ and characterized by amplitude parameters. Mean estimates of bootstrap distributions of temporal parameters and their corresponding CIs were obtained using the same procedure. To facilitate group comparisons, all mean bootstrapped nHRFs were first normalized by each subject’s mean pHRF peak within the V1 SR and then rescaled by group-mean pHRF peak amplitude within the V1 SR across subjects.

For depth-dependent HRF analysis, each ROI was subdivided into nine cortical depth bins spanning normalized depth values from *w* = 0.05 to 0.95 minimizing partial volume effect from pial vasculature and white mater. Voxels were assigned to bins in 0.1-wide intervals, and time series were averaged across voxels within each bin. The same bootstrap procedure was then applied across the 72 events to obtain bootstrapped mean HRFs and 68% CIs for each depth bin. To evaluate depth-dependent changes in the INR and peak amplitude of the nHRF, the TTI and TTP were first identified from the mean bootstrapped nHRF. Amplitude values at these time points were then extracted from each mean bootstrapped nHRF for every depth bin, yielding distributions of INR and peak amplitudes across bootstrap samples. Linear regression was performed on the bootstrap-derived amplitude estimates to test for systematic changes in nHRF’s INR and peak amplitude as a function of cortical depth. Depth-dependent trends were considered significant at *p* < 0.05. Separately, to compare deep and superficial gray matter, we also obtained bootstrapped HRFs and their 68% of CIs for two depth bins (deep: *w* = 0.05–0.35; superficial: *w* = 0.65–0.95) by performing the same procedure. Paired t-tests were performed on bootstrap-derived distributions to assess differences between depth compartments.

## 3. RESULTS

### 3.1 Distance-dependent variations in nHRFs

We observed a systematic change in nHRF temporal dynamics with increasing cortical distance from the SR on the contralateral side, a pattern that was consistently observed across subjects (Fig. 2A). Near the SR, weak but significant INRs were accompanied by relatively large peaks. As distance from the SR increased, INRs became progressively larger in magnitude and more delayed, whereas peak amplitudes decreased and occurred later in time. Although the overall pattern was consistent across subjects, the magnitude and timing of these changes exhibited some inter-individual variability. These findings are consistent with our previous observations^19^.

In more peripheral regions, nHRFs were characterized by strong INRs accompanied by weak or absent peaks, resembling an inverted pHRF as reported previously^4,7,48^. Despite subject-to-subject variability, TTI reached a plateau beyond a certain cortical distance from the SR accompanied by prominent INR, a pattern consistently observed across subjects and visual areas. However, the cortical distance at which the TTI reached the plateau varied substantially among subjects (Fig. 2A) and visual areas (Fig. 2B). For example, in one representative subject, V1 and dorsal V2 TTI reached a plateau within ~ 3 mm of the SR, with a relatively rapid transition to nHRFs characterized by strong INRs and weak peak amplitudes (Fig. 2B). In contrast, dorsal V3 reached the TTI plateau ~ 9 mm from the SR. Interestingly, across subjects and visual areas, we consistently observed two distinct nHRF patterns: one characterized by a significant but weak INR followed by a strong peak amplitude, and the other by a strong INR followed by a weak or absent peak amplitude.

### 3.2 nHRF Categorization

Based on the spatial progression of nHRF temporal dynamics identified in the dilation analysis, we classified nHRFs into two distinct subtypes: transient nHRF (T-nHRF) and fully-developed nHRF (FD-nHRF), Fig. 3A. T-nHRFs exhibit pHRF-like temporal dynamics, characterized by an early, weak but significant INR (CNR > 1) followed by a prominent peak whose amplitude exceeds the absolute INR amplitude (Fig. 3A, green). In contrast, FD-nHRFs are characterized by a delayed, pronounced INR accompanied by a weak or absent peak, such that the absolute INR amplitude exceeds the peak amplitude (Fig. 3A, red).

We first examined how the relative proportions of T- nHRFs and FD-nHRFs varied along the cortical surface. Because the number of dilation ROIs varied with V1 size across subjects, distances from the SR were normalized to each subject’s maximum distance, ranging from 0 at the SR boundary to 1 at the peripheral boundary. The proportions of both nHRF subtypes are shown as a function of normalized distance from the SR within V1 for all subjects, together with the mean (solid line) and standard deviation (shaded regions) across subjects (Fig. 3B). T-nHRFs predominated near the SR and comprised the majority of nHRFs in these regions. Nevertheless, FD-nHRFs were consistently observed at relatively low frequencies. As cortical distance from the SR increased, the proportion of T- nHRFs declined rapidly, whereas FD-nHRFs became progressively more prevalent. Beyond the crossover point (thick vertical magenta line), FD-nHRFs became the dominant subtype throughout most of the peripheral regions. Although inter-subject variability was observed in this trend, the overall shift in subtype proportions with increasing distance from the SR was consistent across subjects (Fig. 3C). T-nHRFs were still observed in far peripheral regions of V1, typically as small and scattered clusters. Notably, the distance at which FD-nHRFs became the predominant response type agreed closely with the distance at which the TTI began to plateau (thick vertical magenta line).

This spatial transition was further examined across visual areas by comparing the cortical distance from the SR at which the TTI began to plateau (Fig. 3D). Across visual areas, the mean transition distance ranged from approximately 3 to 6 mm from the SR. V1 showed a mean distance of 3.4 mm, whereas the mean distances in the extrastriate visual areas were generally ranged from 4 to 6 mm. Substantial inter-subject variability was observed across all visual areas, as reflected by the overlapping error bars.

### 3.3 Regional differences in nHRF parameters and proportions

Based on the spatial transition identified in the dilation analysis, we divided each contralateral visual area into two ROIs: near-SR and peripheral. The boundary was defined by the crossover point between T-nHRF and FD-nHRF proportions, which closely matched the onset of the TTI plateau.

Across visual areas, nHRF parameters in the near-SR region differed from those in the peripheral and corresponding ipsilateral regions, while peripheral and ipsilateral nHRFs showed similar profiles (Fig. 4A). Near the SR, nHRFs showed relatively weak INRs (−0.18 ± 0.04%) with early TTIs (TTI: 2.3 ± 0.27s), together with large peaks (0.48 ± 0.06%) and early TTPs (6.9 ± 0.72s), consistent with T-nHRF characteristics. In contrast, peripheral and ipsilateral nHRFs showed FD-nHRF-like characteristics, with strong INRs and delayed TTIs (peripheral: −0.39 ± 0.05%, 6.5 ± 0.84s; ipsilateral: −0.34 ± 0.04%, 6.8 ± 0.52s) followed by weak and later peaks (peripheral: 0.16 ± 0.04%, 12.5 ± 1.51s; ipsilateral: 0.15 ± 0.02%, 12.4 ± 1.58s).

We examined the differences in nHRF parameters between the near-SR and peripheral ROIs across visual areas (Fig. 4B). We found significant differences in INR amplitude (0.2 ± 0.06%) and TTI (4.1 ± 1.1s) across visual areas. Similarly, peak amplitude and TTP differed between the two regions, with average differences of 0.37 ± 0.19% and 3.3 ± 1.1 s, respectively. Thus, the transition from near-SR to peripheral nHRFs was observed across visual areas, although the magnitude of the parameter changes varied by region.

The regional distribution of nHRF subtypes showed a consistent spatial pattern across subjects (Fig. 5). T-nHRFs were more prevalent in the near-SR ROIs, averaging 70% of all nHRFs across visual areas, whereas their proportions decreased in the peripheral and ipsilateral regions. In contrast, FD-nHRFs were less prevalent near the SR but increased in more distant regions, predominating in both the peripheral (mean: 76%) and ipsilateral (mean: 79%) regions. Although the relative proportions varied across subjects and visual areas, this spatial shift from T-nHRFs near the SR toward FD-nHRFs in more distant regions was consistently observed across the visual cortex.

### 3.4 Depth-dependent variations in nHRFs

We observed depth-dependent changes in the temporal evolution of nHRFs (Fig. 6). In T-nHRFs, both the INR and subsequent peak became more pronounced from deep to superficial gray matter, resulting in larger INR and peak amplitudes near the cortical surface (Fig. 6A). FD-nHRFs also showed a similar depth-dependent pattern (Fig. 6B). The INR exhibited a clear change across cortical depth, becoming progressively larger toward the superficial layers. These depth-dependent changes were observed across subjects, indicating that the temporal profile of both nHRF subtypes varied systematically across cortical depth.

We also quantified these depth-dependent differences by comparing nHRF amplitudes between the predefined deep and superficial gray matter regions in the near-SR, peripheral, and ipsilateral ROIs (Fig. 7). In the near-SR ROI (Fig. 7A), T-nHRF INR amplitude showed significant depth differences in only 2 of 10 subjects, whereas peak amplitude was significantly larger in superficial gray matter in all subjects. FD-nHRF INR amplitude also showed significant depth differences in 4 of 10 subjects. In the peripheral ROI (Fig. 7B), no significant depth differences were observed in T-nHRF INR amplitude, while T-nHRF peak amplitude was significantly larger in superficial gray matter in 4 of 10 subjects. Noticeably, FD-nHRF INR amplitude showed a more consistent depth dependence, with significantly strong amplitudes in superficial gray matter in 9 of 10 subjects. In the ipsilateral ROI (Fig. 7C), significant depth differences in T-nHRF INR and peak amplitudes were observed in 4 and 6 of 10 subjects, respectively, whereas FD-nHRF INR amplitude was significantly stronger in superficial gray matter in 8 of 10 subjects. Overall, these results show that the depth dependence varied with both nHRF subtype and region: the T-nHRF peak showed the most consistent depth effect near the SR, whereas the FD-nHRF INR showed a strong depth effect in the peripheral and ipsilateral regions.

We further examined depth-dependent trends within each ROI using the predefined nine normalized depth bins and found a similar pattern. Significant linear trends were observed predominantly for FD-nHRFs, showing in 8 of 10 subjects in the peripheral ROI and 7 of 10 subjects in both the near-SR and ipsilateral ROIs. In contrast, significant depth-dependent trends were less common for T-nHRFs.

## 4. DISCUSSION

Previously, we demonstrated that the nHRF is not a single stereotyped response but exhibits substantial spatial variability^19^. Using a unilateral visual stimulus with precise retinotopic delineation of the SR, we showed that nHRF temporal dynamics evolve progressively with cortical distance from the SR. In this study, we expanded both the experimental design and the analysis to characterize the nHRF in substantially greater detail. First, by relocating the SR to the parafoveal representation (1–3° eccentricity, compared with 4–6° previously), we preserved longer continuous cortical regions outside the SR, which allowed us to track the temporal evolution of the nHRF continuously from the SR to the peripheral representation. On this basis, we defined two nHRF subtypes: a transient nHRF (T-nHRF) with early and weak INRs followed by prominent peaks, and a fully developed nHRF (FD-nHRF) characterized by delayed and strong INRs with weak or absent peaks. Second, we showed that the two nHRF subtypes are not spatially segregated but coexist throughout visual cortex in regionally varying proportion. T-nHRFs predominate near the SR, with the dominant subtype shifting toward FD-nHRFs at greater distances on the contralateral side. FD-nHRFs likewise predominate in homologous ipsilateral regions. Third, we examined the cortical-depth dependence of each nHRF subtype, which has not been characterized previously. In T-nHRFs, depth dependence was shown mostly in subsequent peak, which increased toward superficial gray matter most consistently in the near-SR regions, whereas in FD-nHRFs, it was shown primarily in the INR, which increased in magnitude toward superficial gray matter most consistently in peripheral contralateral and ipsilateral regions. Notably, each nHRF subtype exhibited its most consistent depth dependence in the regions where it predominated. Together, these findings extend our previous observations by establishing that nHRFs are systematically organized along both cortical distance from the SR and cortical depth, providing a framework for interpreting the spatiotemporal characteristics of negative BOLD responses.

In our previous work, the contralateral nHRF appeared to undergo a progressive temporal evolution with cortical distance from the SR^19^, which we interpreted as a gradual transformation of a single response type. However, in this study, we found two temporally distinct nHRF subtypes that coexist throughout the visual cortex, with their relative proportions shifting along the cortical surface (Figs. 3&5). The temporal progression observed in band ROI-averaged nHRFs (Fig. 2) therefore can reflect two co-occurring changes—the shifting proportions of the two nHRF subtypes and more gradual changes in temporal characteristics within each subtype.

The origins of the NBR have been debated over a decade. Vascular redistribution, often termed “blood steal”^4,14^, and neural suppression^11,15^ have been proposed, and evidence against each has also been reported^49,50^. Our results can raise the possibility that the two mechanisms are not mutually exclusive but coexist. Our hypothesis is that the T-nHRF mainly reflects vascular redistribution while the FD-nHRF reflects neural suppression. Blood steal should be confined to the vicinity of the SR, following dynamics of the adjacent positive response without changing local CMRO_2_, which would be consistent with T-nHRFs predominated near the SR, showing early and weak INRs followed by a positive peak^51^. This may indicate passive recovery of local perfusion after the initial deficit. The peak of the T-nHRF also became larger toward superficial gray matter, as reported for the pHRF peak^28,52^, which would be consistent with a flow-mediated origin expressed through the vascular architecture (see below for more details). The FD-nHRF, in contrast, may instead reflect neural mediated suppression; a strong INR with a minimal or absent peak would be consistent with neurovascular coupling to reduced neural activity^11,15^. It predominantly occurs in peripheral and ipsilateral regions where no adjacent positive response is available for blood redistribution. In addition, the temporal dynamics and subtype proportions of peripheral and ipsilateral nHRFs were similar, consistent with a common suppressive origin^7,53^. Our recent multi-contrast laminar fMRI study at 7T found concurrent reductions in CBF and CMRO_2_ in visual representations remote from the SR^54^, supporting a suppressive origin for negative response arising away from the PBR. Together, we suggest that the two nHRF subtypes may reflect the coexistence of vascular redistribution and neural suppression dominating different spatial regimes.

The two-subtypes framework also provides a natural interpretation of the TTI plateau, which would be difficult to explain with a single stereotypical nHRF changing gradually with cortical distance from the SR. Because each dilation-band ROI averages the two different nHRF subtypes in different proportions, the band-averaged TTI shifts later as the FD-nHRF proportion grows and stabilizes once FD-nHRFs dominate; accordingly, the onset of the TTI plateau roughly corresponds to the crossover point between the two nHRF subtypes’ proportions. Both the shift in subtype proportion and distance-dependent change in the individual subtype, particularly that of the T-nHRF, appear to drive the apparent temporal evolution of the band-averaged HRFs.

In contrast to the well-established depth dependence of the pHRF^28,29^, the depth dependence of the NBR has been inconsistent across previous studies^3,54–56^; in particular, the depth dependence of the nHRF has remained largely unexamined. The nHRF—NBR evoked by a brief stimulus—can offer a cleaner window than the sustained NBR. A long stimulus does not necessarily evoke a temporally stable neural response; phenomena such as variable attention and adaptation can unpredictably modulate the response, making it difficult to resolve the details of the physiological responses^57,58^. A brief stimulus also makes the underlying physiology simpler because the BOLD signal can be attributed primarily to CBF and CMRO_2_ changes. We can exclude BOLD changes introduced by CBV changes because there is no significant change in CBV for a brief stimulus based on previous experiments^59^. With this approach, we examined the depth dependence of each nHRF subtype separately and found that the two subtypes exhibited distinct depth dependencies. In T-nHRFs, the depth dependence appeared in the peak following the INR rather than the INR itself. This would be consistent with the vascular redistribution: blood is diverted at the level of the penetrating arterioles toward the activated SR, and the subsequent peak of the T-nHRF reflects the recovery of local inflow. As a consequence, this flow-driven transient is expressed downstream through the same ascending venules and pial veins, whose drainage weighs the BOLD signal toward the surface, explaining the superficial increase of the peak of the T-nHRF. In FD-nHRFs, the depth dependence appeared in the INR itself, indicating that this negative signal—most likely suppression-driven—is amplified toward superficial gray matter through the same venous drainage, occurring in both the peripheral contralateral and ipsilateral regions. Notably, globally distributed FD-NHRFs on the ipsilateral side, where suppression is driven remotely, showed the same depth dependence as those in peripheral contralateral regions, which may indicate a common suppressive origin for the FD-nHRF in both regions^7^.

These subtype-specific profiles make it important to analyze each subtype within the regions where it predominates. A simple averaging scheme can obscure the actual depth dependence of the nHRF. For example, near the SR, the INR of the FD-nHRF and the subsequent peak of the T-nHRF occur at similar latencies, so averaging them can diminish or abolish the apparent negative response. Such cancellation may underlie some of the weak or absent depth dependence of the NBR reported previously.

Despite the dominance of each nHRF subtype in different regions, the two subtypes were not clearly segregated: a minority of far peripheral and ipsilateral voxels were classified as T-nHRFs while FD-nHRFs were consistently observed near the SR. Several factors may contribute to these minor responses. Defining the different subtypes relies on a threshold, which creates ambiguity for nHRFs close to the threshold. In addition, despite the evident crossover point and TTI jump, a transition region between two ROIs (near SR and peripheral) on the contralateral side can exist, where both subtypes are present. For T-nHRFs in peripheral contralateral and ipsilateral regions, the CNR of nHRFs was relatively low (i.e., noise was strong relative to the INR and peak amplitude), which can make subtype classification less reliable. This may explain the scattered patterns of these minor responses. These factors, however, may not fully account for the observations. Strong negative responses near the SR may be compatible with suppressive processes, as reported in previous studies^15^. T-nHRFs found in peripheral contralateral and ipsilateral regions may also be genuine, but they cannot be attributed to vascular redistribution. One possibility is that the negative response varies strongly across cortical depth in some local regions. For example, if a locally weak negative response in deep gray matter while transient response is retained in the superficial gray matter, the depth-averaged response would resemble a T-nHRF.

The study has several limitations. First, although we carefully delineated the signal from the SR using a retinotopic mapping to minimize partial volume effects, some of the T-nHRFs near the SR may reflect signal spread from the positive response rather than a change in local perfusion. Second, the effect of attention has not been characterized. In this study, the task demanded attention to the stimulated location. Attention was therefore strongly focused on the stimulus, while the surrounding and ipsilateral representations were unattended. However, because this attentional state was fixed, we could not assess how attention affects the temporal dynamics of the nHRF. Future work may be required to characterize the effect of the attention on the temporal dynamics of the nHRF. Third, the two different nHRF subtypes defined in this work are limited to visual cortex. Future works need to examine whether these temporal features of the nHRF hold in other sensory and association cortices. Finally, although our findings provide a detailed characterization of nHRF temporal dynamics, they are based solely on BOLD fMRI and thus cannot directly distinguish between the underlying neural and vascular mechanisms. Simultaneous measurements of CBF, CMRO_2_, and neural activity using complementary techniques such as arterial spin labeling (ASL), calibrated fMRI, or electrophysiological recordings will provide a framework to distinguish the physiological mechanisms underlying transient and FD nHRFs and to test the mechanistic hypotheses proposed here.

## Figures and Tables

**Figure 1:**
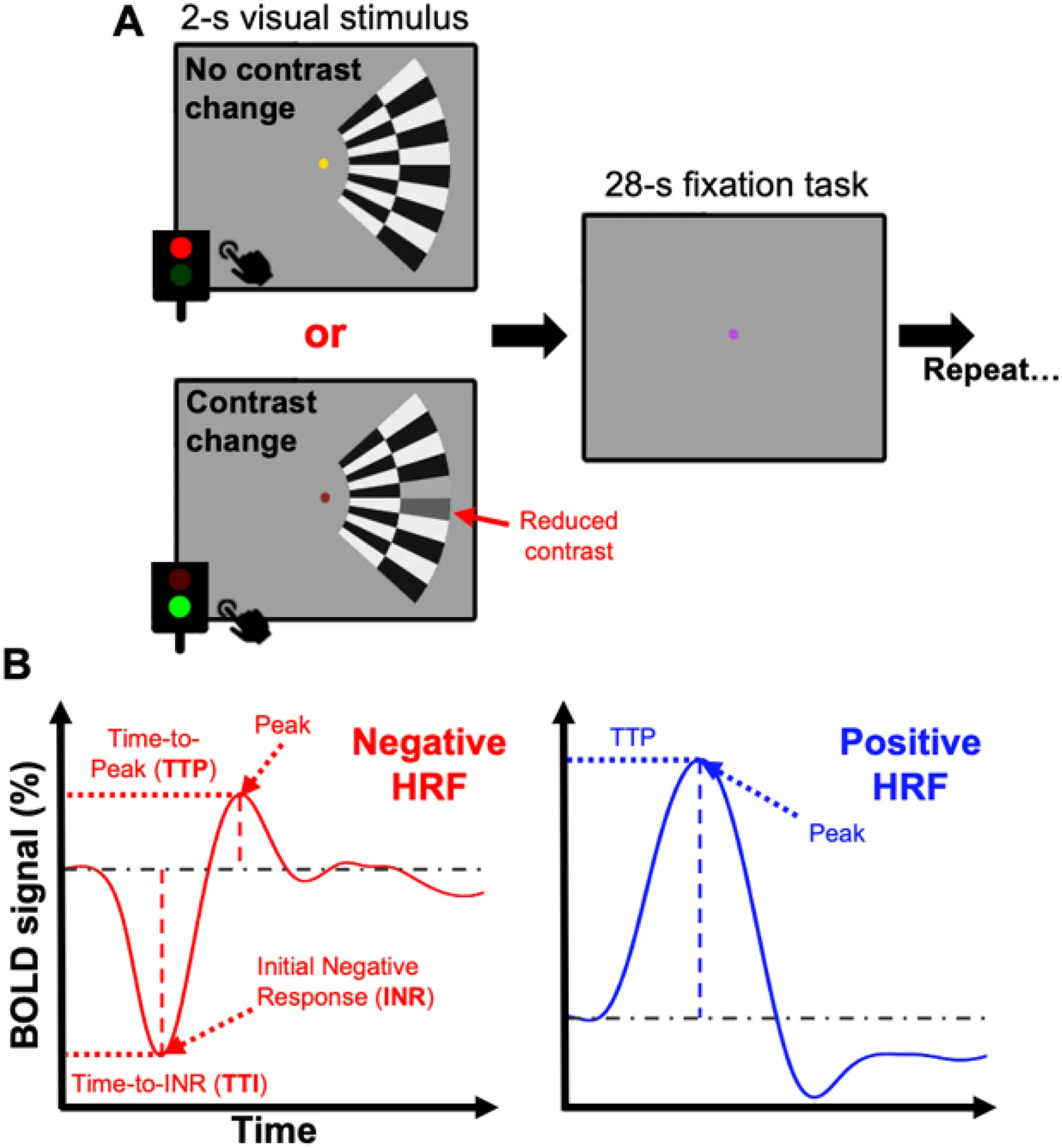
**A**) Example of the partial unilateral stimulus consisting of 10 sectors at eccentricity 1–3° and polar angle 50–130°. A random sector within the stimulus either exhibited a contrast change or remained unchanged, followed by a 28-s fixation-only task. **B**) Example of negative (left) and positive (right) HRFs with their parameters.

**Figure 2:**
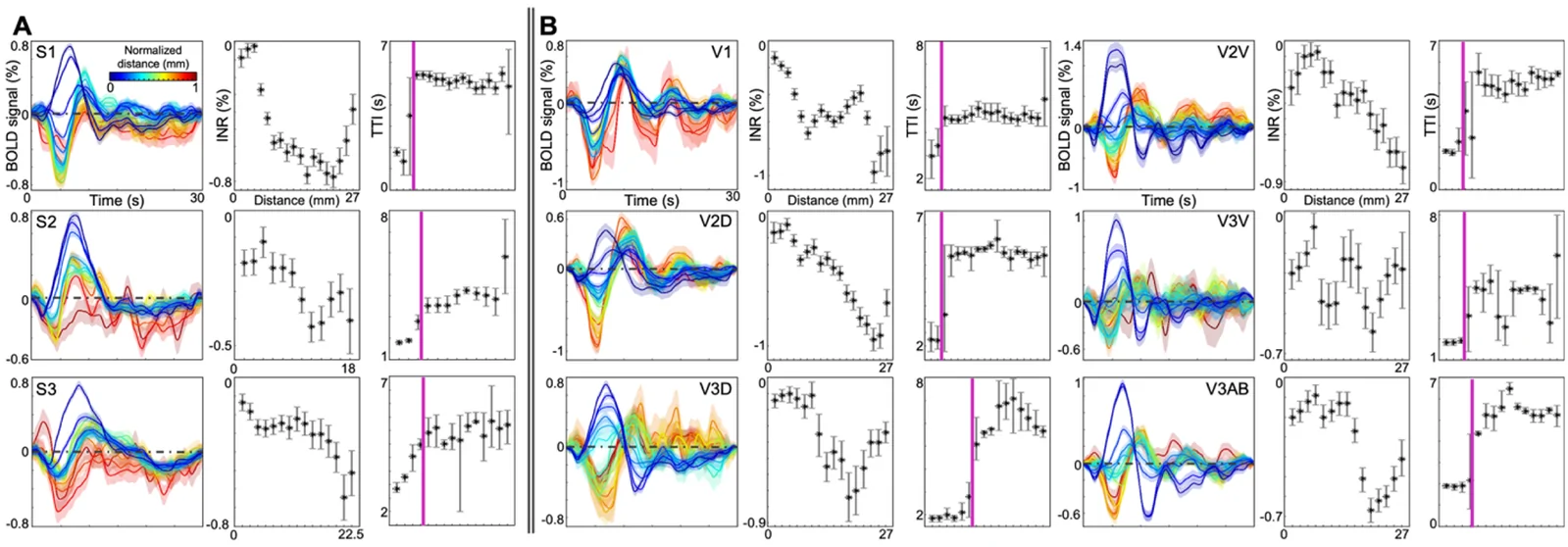
Changes in nHRF temporal dynamics and INR/TTI parameters across 1.5-mm-wide ROIs dilated outwards from the SR **A**) for three subjects in V1 and **B**) for a representative subject in different ROIs: V1, dorsal V2/V3, ventral V2/V3, and V3AB. Shaded areas and error bars indicate 68% confidence intervals. The thick purple line marks the point at which the TTI begins to plateau.

**Figure 3:**
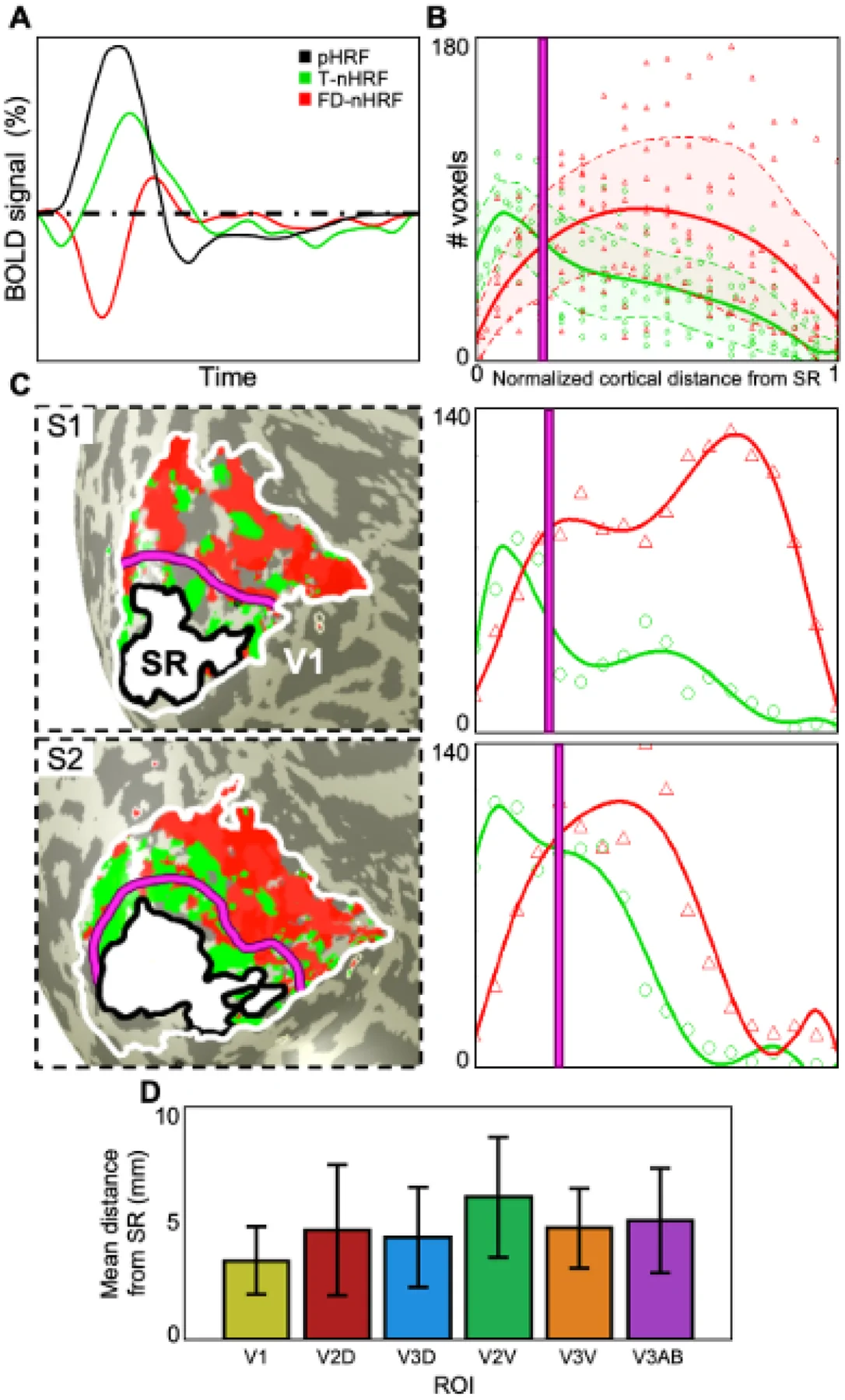
**A**) Temporal dynamics of pHRF and two nHRF subtypes: transient nHRF (T-nHRF) and fully developed nHRF (FD-nHRF). **B**) Distribution of both subtypes of nHRFs across all subjects relative to the normalized cortical distance outwards from the SR within V1. Solid lines indicate the average proportion across all subjects and shaded areas indicate 68% confidence intervals. The thick purple line shows where the percentage contribution of T-nHRFs intersects with that of FD-nHRFs. **C**) Spatial patterns of both nHRF subtypes in V1 for two representative subjects (left) and their corresponding distributions relative to the normalize distance from the SR (right). The thick purple line shows the distance at which the TTI plateau begins. D) The average distance across subjects for the beginning of the TTI plateau in V1, dorsal V2/V3, ventral V2/V3, and V3AB ROIs.

**Figure 4:**
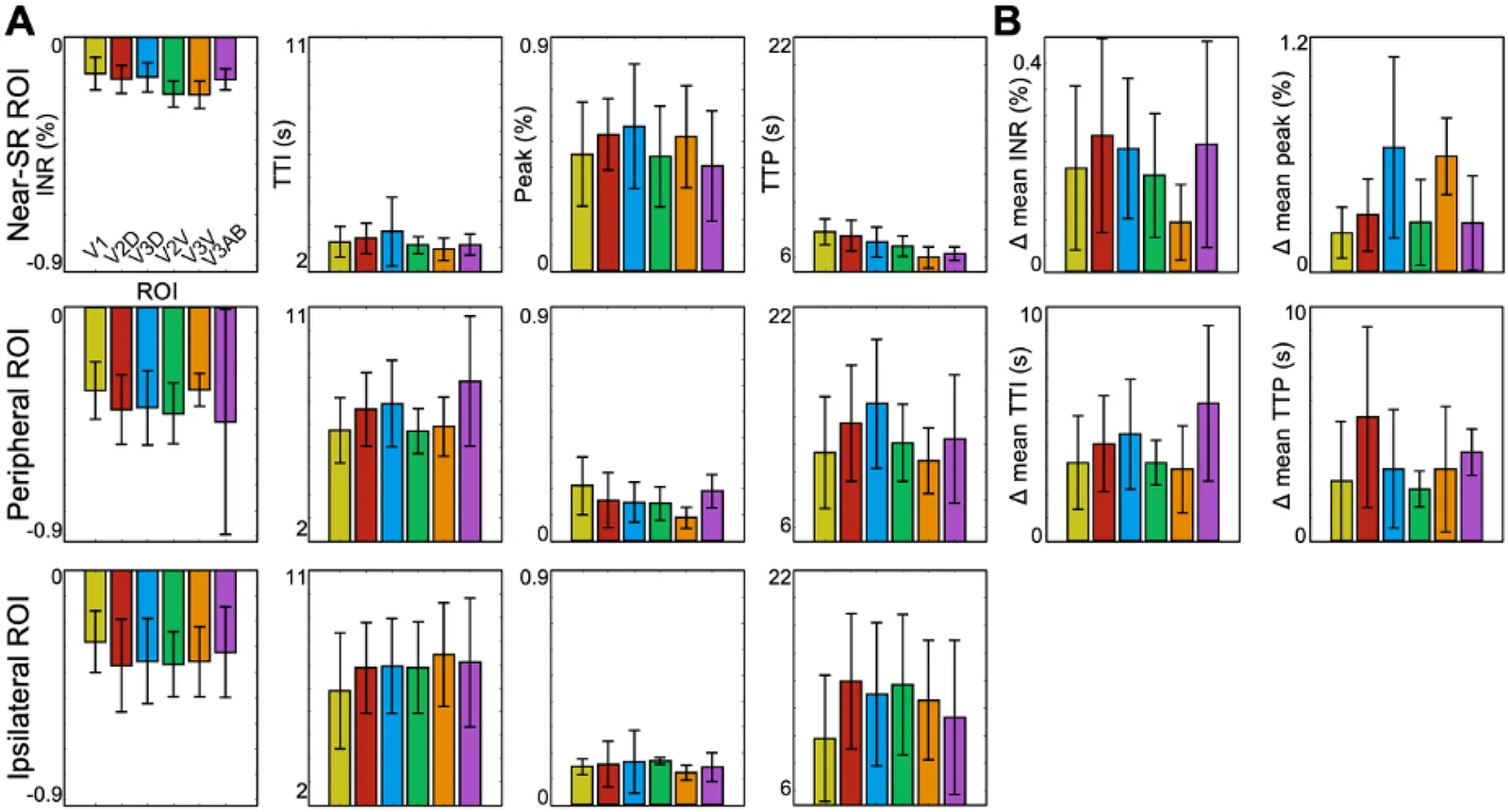
**A**) nHRF parameter plots averaged across all subjects for V1, dorsal V2/V3, ventral V2/V3, and V3AB regions within three different areas: near-SR, peripheral, and ipsilateral ROIs. **B**) The difference in each parameter between near-SR and peripheral ROIs averaged across all subjects within all visual areas. Error bars indicate 68% confidence intervals.

**Figure 5:**
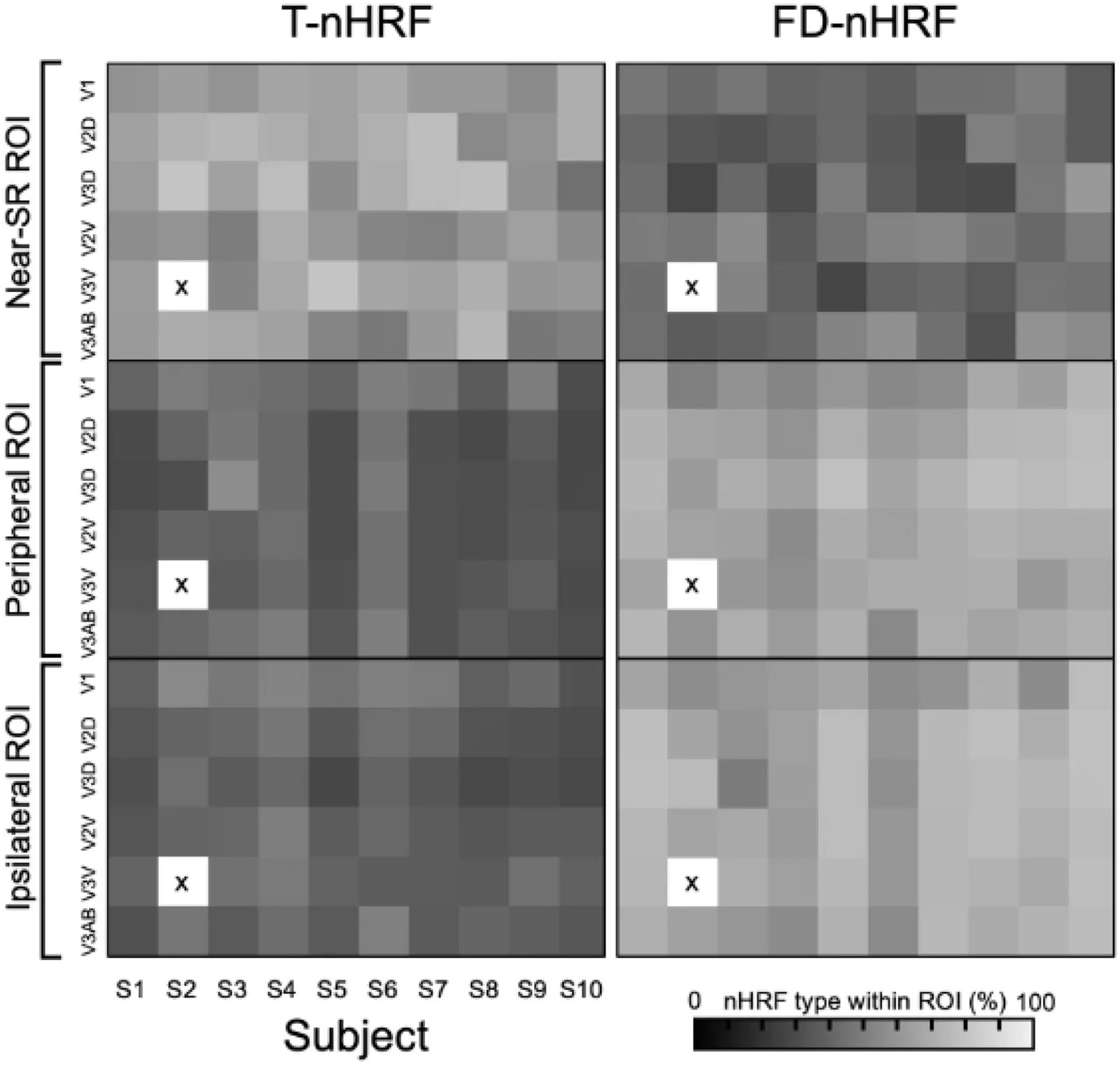
Heatmaps for the proportion of T-nHRFs (**left**) and FD-nHRFs (**right**) across all subjects in the near SR, peripheral, and ipsilateral ROIs within V1, dorsal V2/V3, ventral V2/V3, and V3AB regions. Lighter colors indicate higher proportions in those regions. White areas marked with “x” indicate the absence of that ROI.

**Figure 6:**
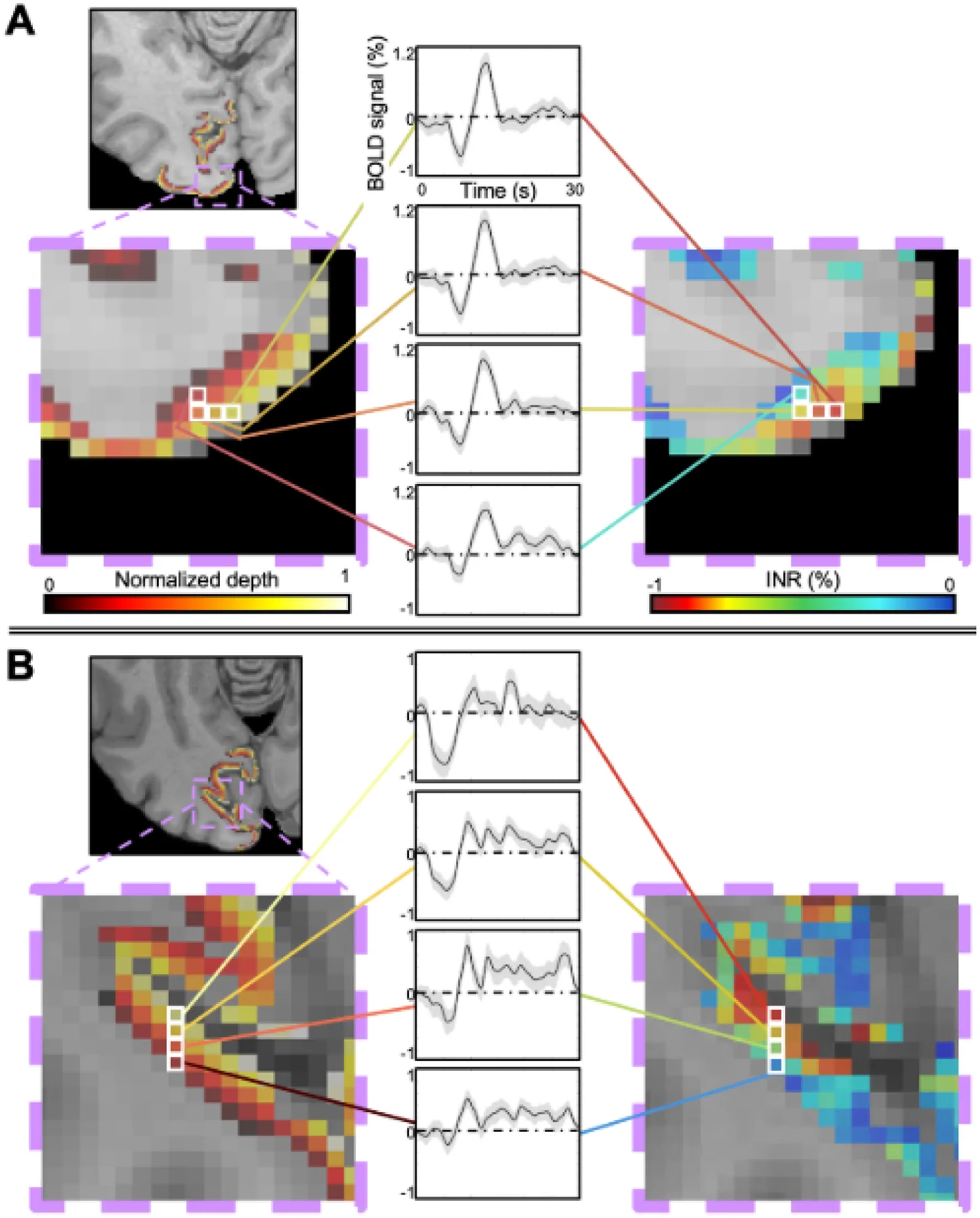
Normalized depth and INR parameter maps alongside nHRF time series in four voxels within V1 from deep (lower) to superficial (upper) gray matter shown for two representative subjects. Changes across cortical depths are highlighted for **A**) T-nHRFs and **B**) FD-nHRFs. Shaded gray region indicates 68% confidence intervals.

**Figure 7:**
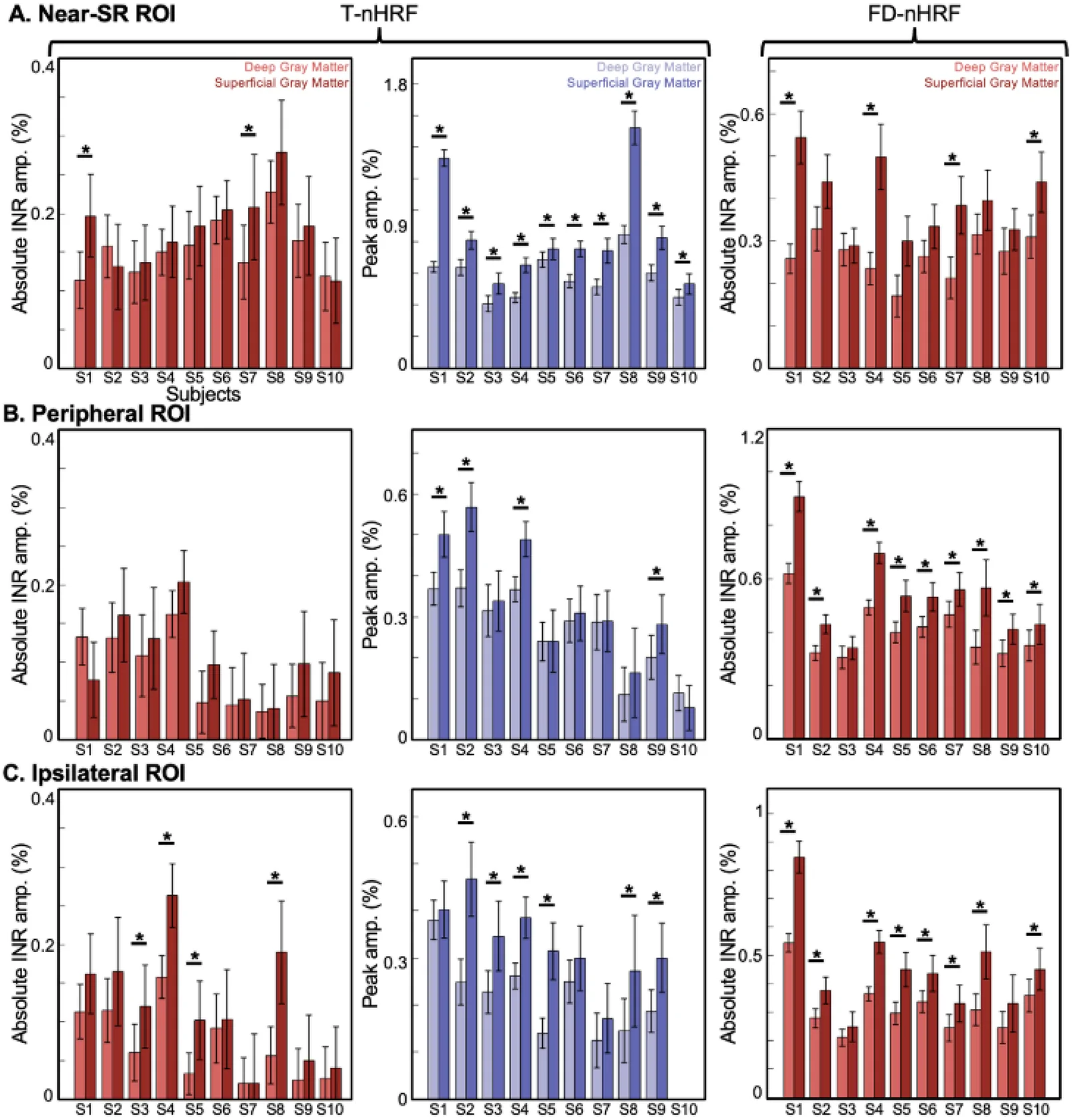
Mean INR and peak amplitudes extracted from T-nHRFs and INR amplitudes extracted from FD-nHRFs for each subject in deep and superficial depths within V1, shown for the near SR (A), peripheral (**B**), and ipsilateral (**C**) ROIs. Error bars indicate 68% confidence intervals. Asterisks denote significant differences between depths with paired t-tests (p < 0.05).

## Data Availability

The raw data supporting the conclusions of this article will be made available by the authors, without undue reservation.
